# Enhancement of neuroprotective activity of Sagunja-tang by fermentation with lactobacillus strains

**DOI:** 10.1186/s12906-018-2361-z

**Published:** 2018-11-28

**Authors:** Nam-Hui Yim, Min Jung Gu, Hee Ra Park, Youn-Hwan Hwang, Jin Yeul Ma

**Affiliations:** Korean Medicine (KM) Application Center, Korea Institute of Oriental Medicine (KIOM), 70 Cheomdan-ro, Dong-gu, Daegu, 701-300 Republic of Korea

**Keywords:** Sagunja-tang, Neuroprotective effect, Neuroblastoma, *Lactobacillus plantarum* 166, Phytochemicals

## Abstract

**Background:**

Sagunja-tang (SGT) is widely used in traditional herbal medicine to treat immune system and gastrointestinal disorders and reportedly has protective effects against inflammation, cancer, and osteoporosis. In this study, we fermented SGT with different *Latobacillus* strains and investigated the change in phytochemical compositions in SGT and enhancement of it neuroprotective effects in SH-SY5Y human neuroblastoma.

**Methods:**

Marker components, including ginsenoside Rg_1_, glycyrrhizin, liquiritin, liquiritigenin, atractylenolide I, atractylenolide II, atractylenolide III, and pachymic acid, in SGT, were qualitatively and quantitatively analyzed using high-performance liquid chromatography–diode array detection and liquid chromatography–mass spectrometry. SGT was fermented with eight different *Lactobacillus* strains to yield eight fermented SGTs (FSGTs). The conversion efficiencies of SGT marker components were determined in each FSGT. To detect the protective effect of SGT and FSGT, reactive oxygen species (ROS) assay and mitochondrial membrane potentials (MMPs) assay were performed in SH-SY5Y cells.

**Results:**

Compared with the other FSGTs, SGT166, i.e., SGT fermented with *L. plantarum* 166, had high conversion efficiency, as indicated by increased amounts of glycyrrhizin, liquiritigenin, and atractylenolides I–III. In SH-SY5Y cells, protection against cell death induced by H_2_O_2_ and etoposide was high using SGT166 and very low using SGT. Furthermore, ROS production and mitochondrial membrane potential disruption in SH-SY5Y cells were markedly suppressed by SGT166 treatment, which demonstrated that inhibition of ROS generation may be one of the neuroprotective mechanisms of SGT166.

**Conclusions:**

This study demonstrated that fermentation of SGT with *L. plantarum* 166 enhanced suppression of oxidative stress and MMP loss. This enhanced neuroprotective effect was thought to be caused by the conversion of SGT phytochemicals by fermentation. SGT166 shows potential for treating neurological damage-related diseases.

**Electronic supplementary material:**

The online version of this article (10.1186/s12906-018-2361-z) contains supplementary material, which is available to authorized users.

## Background

Sagunja-tang (SGT; Sijunzi-tang in Chinese and Shikunshi-to in Japanese) is a famous traditional herbal medicine in eastern Asia that has been used to treat spleen deficiency and gastrointestinal disorders, such as vomiting, diarrhea, and ulcer [1]. Recently, SGT has been shown to confer a variety of pharmacological activities, including anti-inflammation, anti-cancer, and anti-ulceration activities and regulation of bone metabolism [2–5]. SGT is composed of ginseng radix, atractylodis rhizoma, poria sclerotium, and glycyrrhizae radix at a ratio of 1:1:1:1, which themselves contain various phytochemicals, including ginsenoside (ginseng radix), atractylenolides I–III (atractylodis rhizoma), pachyman and pachymic acid (poria sclerotium), liquiritin, liquiritigenin, and glycyrrhizin (glycyrrhizae radix). Recently, Kang et al. investigated simultaneous analytical methods suitable for quantifying the major compounds in SGT [6]. In our study, their established method was modified and applied to analyze seven marker compounds in SGT and fermented SGT (FSGT).

Fermentation with probiotics, such as *Lactobacillus* spp., *Bifidobacterium* spp., and *Saccharomyces* spp., has been shown to affect the conversion of phytochemical composition and improve the therapeutic effects of some herbal medicines [7–9]. The results of several studies have suggested that fermentation of natural medicines with *Lactobacillus* increases the concentrations of bioactive components, especially antioxidants, which influence bioactivities, including antiinflammatory, antiosteoporotic, and anticancer activities [10–13]. Our previous study identified the antiamnesic effects of Sipjeondaebo-tang fermented with *Lactobacillus* in a scopolamine-induced amnesia mouse model [14].

In neuronal cells, oxidative stress causes mitochondrial dysfunctions, which is involved in apoptotic cell death. Reactive oxygen species (ROS) and reactive nitrogen species (RNS) play an important role as regulatory mediators in physiological system, however, the high concentrations of these species are involved in cellular damage, which causes the neurodegenerative diseases, including Alzheimer’s disease (AD), Parkinson’s disease (PD), Mutiple Sclerosis (MS) and Amyotrophic lateral sclerosis (ALS) [15–19]. Especially, ROS are active in the brain and neuronal tissue and generally lead to neuronal damage and apoptotic cell death induced by intracellular micro-environmental changes [20]. Hence, inhibiting ROS generation can be a useful way to protect normal neuronal cells from damage or death that leads to neurodegenerative diseases.

In the present study, we investigated the conversion of phytochemicals caused by fermentation of eight batches of SGT with eight strains of *Lactobacillus.* We then assessed the neuroprotective effects of SGT alone and of SGT fermented with *L. plantarum* 166 (SGT166) in SH-SY5Y human neuroblastoma.

## Methods

### Chemicals and reagents

**Fig. 1:**
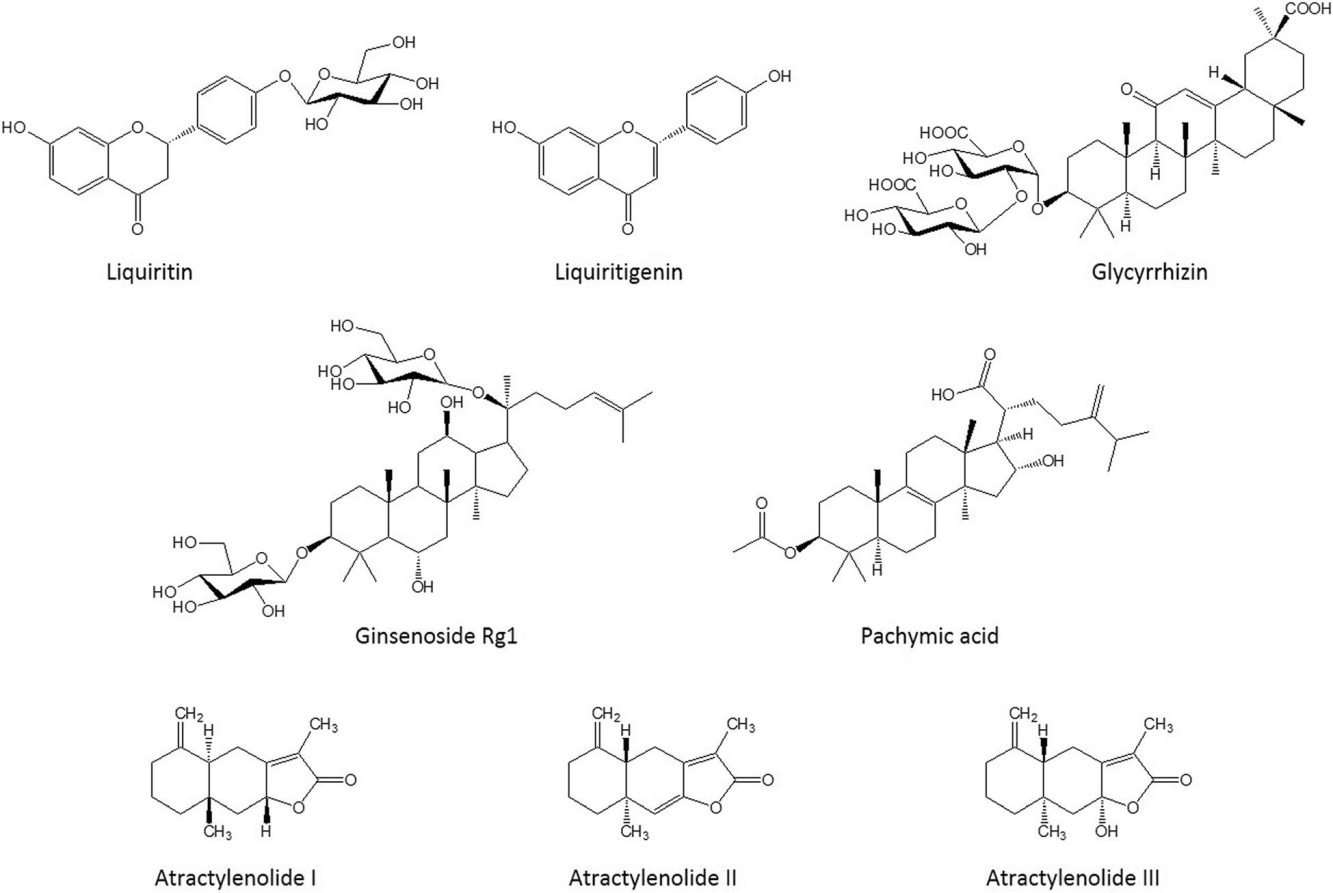
Chemical structures of the eight components in SGT

Ginsenoside Rg_1_ and glycyrrhizin were purchased from the Korea Food and Drug Administration and Tokyo Chemical Industry Co., Ltd. (Tokyo, Japan), respectively. Liquiritin, liquiritigenin, atractylenolide I, atractylenolide II, atractylenolide III, and pachymic acid were purchased from Faces Biochemical Co., Ltd. (Wuhan, China). The purities of these seven reference standards were > 98%, and their structures are shown in Fig. 1. Trifluoroacetic acid (TFA) and formic acid were purchased from Sigma-Aldrich Co. (St. Louis, MO, USA). Acetonitrile of high-performance liquid chromatography (HPLC) grade was obtained from J.T. Baker Inc. (Philipsburg, NJ, USA), and deionized water was prepared using an ultrapure water production apparatus (Millipore, Billerica, MA, USA).

### Preparation of SGT and FSGT

All herbal constituents of SGT, including ginseng radix (KMAC112), atractylodis rhizome (KMAC059), poria sclerotium (KMAC061), and glycyrrhizae radix (KMAC003), were purchased from the Korea Medicinal Herbs Association (Yeongcheon, Korea). The origins of the samples were confirmed taxonomically by Prof. Ki Hwan Bae of the College of Pharmacy, Chungnam National University. All voucher specimens were deposited in the herbal bank of the Korean Medicine Application Center, Korea Institute of Oriental Medicine. Four kinds of medicinal herbs (2 kg; Table 1) were extracted into 20 L of water for 3 h using a COSMOS-660 extractor (Kyungseo Machine Co., Incheon, Korea). The extract was filtered through standard testing sieves (150 μm) and freeze-dried to yield SGT extract powder (240 g). To ferment SGT, the following eight bacterial strains were used to generate eight batches: *Lactobacillus rhamnosus* KFRI127, *L. zeae* KFR129, *L. rhamnosus* KFRI144, *L. acidophilus* KFRI150, *L. fermentum* KFRI162, *L. plantarum* KFRI166, *L. acidophilus* KFRI217, and *L. helveticus* KFRI341. The strains were obtained from the Korea Food Research Institute (Seongnam, Korea) and were cultured in de Man, Rogsa, and Sharpe broth at 37 °C for 24 h and reinoculated to each broth under the same conditions. The solutions of SGT extract were adjusted to pH 8.0 with 1 M NaOH and autoclaved for 15 min at 121 °C for use as the culture media in the bacterial fermentations. The solutions were inoculated with 5 mL of the inoculum (1% *v*/v, 2 × 10^9^ CFU/mL). The inoculated samples were incubated at 37 °C for 48 h. The FSGT was lyophilized at 4 °C after filtering through a 60-μm nylon net filter (Millipore, Billerica, Mass, USA).

**Table 1:** Composition of Sagunja-tang (SGT) preparation

Herbal medicine	Amount (g)	Ratio (%)
Ginseng Radix	500	25
Atractylodis Rhizoma	500	25
Poria Sclerotium	500	25
Glycyrrhizae Radix	500	25
Total	2000	100

### Preparation of standard solutions and analytical samples

Individual stock solutions of liquiritin (400 μg/mL), ginsenoside Rg_1_ (500 μg/mL), liquiritigenin (200 μg/mL), glycyrrhizin (900 μg/mL), atractylenolide I (200 μg/mL), atractylenolide II (200 μg/mL), and atractylenolide III (200 μg/mL) dissolved in methanol were mixed and diluted serially to five concentrations for use in generating calibration curves. Quality control samples for method validation were diluted serially to three concentrations. The SGT and FSGTs for quantitative analysis were dissolved at a concentration of 20 mg/mL in methanol. The samples were filtered through a 0.2 μm membrane filter (Whatman International Ltd., Maidstone, UK) and stored at 4 °C before use.

### Chromatographic conditions

The analysis was performed on an HPLC system (Hitachi High-Technologies Co., Tokyo, Japan), consisting of a pump (L-2130), an auto sampler (L-2200), a column oven (L-2350), and a diode array U*V*/VIS detector (L-2455). The system control and data analyses were executed by an EZchrom Elite software (version 3.3.1a) system. The separation was performed on a RS tech C_18_ column (5 μm, 250 × 4.60 mm) with a flow rate of 1.0 mL/min and a column oven temperature of 40 °C. The mobile phase consisted of 0.1% trifluoroacetic acid in water (A) and acetonitrile (B). To improve the chromatographic separation capacity, the gradient elution system was performed as follows: 90–65% A, 0–25 min; 65–55% A, 25–40 min; 55–30% A, 40–55 min; 30% A, 55–65 min. The sample injection volume was 20 μL.

Liquid chromatography/mass spectrometry (LC/MS) analysis was performed on a Dionex UltiMate 3000 system (Dionex Corp., Sunnyvale, CA, USA) equipped with a Thermo Q-Exactive mass spectrometer (Thermo Fisher Scientific, Bremen, Germany). The separation was performed on an Acclaim RSLC 120 C18 column (150 × 2.1 mm, 2.2 μm, Dionex Corp.). The mobile phase consisted of acetonitrile (reservoir A) and 0.1% *v*/v formic acid in water (reservoir B). Gradient elution was as follows: 3% A, 0.0–2.0 min; 3.0–40.0% A, 2.0–15.0 min; 40.0–95.0% A, 15.0–23.0 min; 95.0–95.0% A, 23.0–24.0 min; 95.0–100% A, 15.0–17.0 min; 5% isocratic A, 17.0–22.0 min. The flow rate was 0.4 mL/min. The operation conditions in the MS analysis were set as follows: ionization mode, positive; spray voltage, 4.0 kV; capillary temperature, 320 °C; sheath gas pressure, 40 arbitrary units; auxiliary gas pressure, 10 arbitrary units; ion scans, 100–1500 m/z; resolution of MS scans, 70,000. The SGT or SGT166 (10 mg/mL) and mixture of authentic standards (10 μg/mL) were prepared in methanol and filtered through a 0.22 μm filter membrane before injecting 5-μL aliquots for HPLC–MS analysis.

### Method validation

Method validation for selectivity, linearity, limit of detection (LOD), limit of quantification (LOQ), precision and accuracy, and recovery in the present study was executed according to the guideline of International Conference on Harmonization (ICH).

The standard calibration curve for the linearity assay was prepared with five different concentrations of diluted standard solutions and performed in triplicate independently. The calibration curves were constructed by plotting the value of the peak versus the concentration of each analyte. The lower LOD and lower LOQ for each analyte were determined on the basis of signal-to-noise ratios (S/N) of 3.3 and 10, respectively.

The precision of the analytical method was evaluated by intra- and inter-day tests. Standard solutions at three different concentrations (low, medium, and high) were analyzed. The intra-day test was performed by analyzing a mixed standard solution in five replicates during one day. The inter-day test was performed by analyzing five replicates of the same standard solution on each of three consecutive days. Precision was expressed as relative standard deviation (RSD, %), which is generally acceptable within 3%. The related equation was as follows: RSD (%) = [standard deviation (SD)/mean measured amount] x100. The recovery tests were performed to evaluate the accuracy of the method. The recoveries of analytes were determined by adding three different concentrations of each standard solution into the SGT solution (10 mg/mL) in triplicate. Recovery (%) was calculated according to the following equation: Recovery (%) = [found amount – original amount]/spiked amount x 100.

### Cell culture

SH-SY5Y cells, a human neuroblastoma-derived cell line with neuron-like characteristics, were provided by Prof. Jaewon Lee, Pusan National University, Korea. The cells were differentiated into neuron-like phenotype by induction of retinoic acid and cultured with RPMI 1640 media (Lonza, Walkersville, MD, USA) with heat-inactivated 10% fetal bovine serum (HyClone Laboratories, Utah, USA), 2 mM glutamine, and 1% penicillin/streptomycin antibiotic mixture (Corning Incorporated, NY, USA) in a humidified 5% CO_2_ incubator at 37 °C.

### Cell viability analysis

Cells (1 x 10^4^ cells/mL) were seeded into 96-well plates. After 24 h, the cells were pretreated with different concentrations of samples (250 and 500 μg/mL) for 6 h and co-treated with 50-μM of the toxicants H_2_O_2_ and etoposide for 24 h. After treatment, cell viability was analyzed using a Cell Counting Kit-8 (CCK-8) solution (Dojindo Laboratories, Kumamoto, Japan). Color intensity was measured at 450 nm using ELISA microplate reader.

### ROS production

ROS products were measured using a fluorogenic dye 2′, 7′-dichlorodihydrofluorescein. diacetate (H_2_-DCFDA), which is oxidized by intracellular ROS. Cells (5x10^3^ cells/well) were seeded into black 96-well plates and pretreated with vehicle or samples for 6 h, treated with 50 μM H_2_-DCFDA for 30 min, and then incubated with 50 μM H_2_O_2_ for 30 min. The cells were washed with phosphate-buffered saline, and fluorescent compounds were detected using a fluorescence microplate reader (SpectraMax i3; Molecular Devices, CA, USA) with excitation and emission wavelengths of 495 nm and 529 nm, respectively.

### Mitochondrial membrane potentials (MMPs) assay

Cells were seeded into a confocal dish (coverglass-bottom dish). After pretreatment with vehicle or samples for 6 h, the cells were co-treated with 100 μM H_2_O_2_ for 1 h. The cells were further incubated with JC-1 (chloride salt; Biotium, Hayward, CA, USA) staining solution (5 μg/mL) at 37 °C for 15 min and rinsed with culture media. MMPs were estimated by measuring the fluorescence of free JC-1 monomers (green) to JC-1 aggregates in mitochondria (red) as observed through a Nikon ECLIPSE TE2000-U microscope (Nikon Instruments Inc., Tokyo, Japan), and the quantification of the red and green fluorescence intensity ratio in individual cells was performed using Nikon NIS-Elements microscope imaging software.

### Statistical analysis

Data are presented as means ± SDs. Statistically significant differences between vehicle, toxicants, and sample treated cells were calculated by one-way analysis of variance with Dunnett’s test. The analyses were performed using GraphPad PRISM software ® (GraphPad PRISM software Inc., Version 5.03, CA, USA). Values of *p* < 0.05 were considered as indicating statistical significance.

## Results

### Optimization of chromatographic conditions

**Fig. 2:**
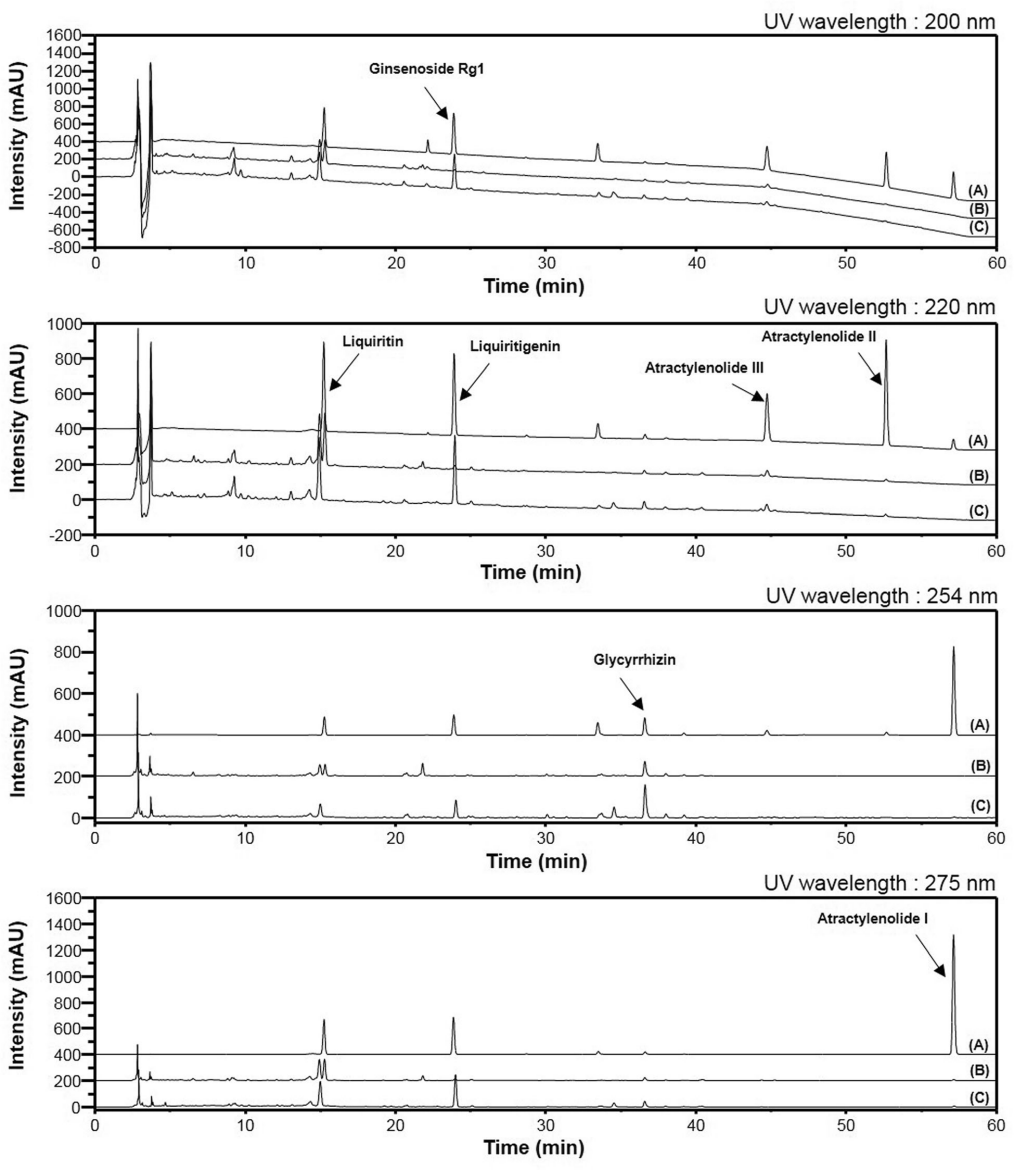
HPLC-DAD chromatogram of standard mixture (**a**), SGT (**b**) and SGT166 (**c**)

**Fig. 3:**
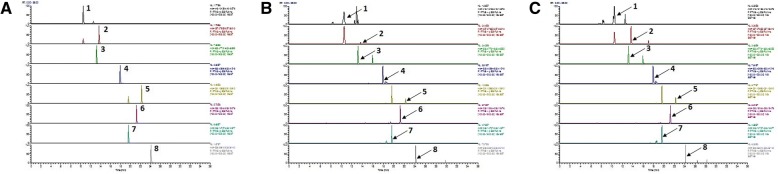
Extracted ion chromatogram (EIC) of standard mixture (**a**), SGT (**b**) and SGT166 (**c**) using UPLC-ESI-MS analysis. **1**, Liquiritin; **2**, Liquiritigenin; **3**, Ginsenoside Rg_1_; **4**, Glycyrrhizin; **5**, Atractylenolide Ι; **6**, Atractylenolide II; **7**, Atractylenolide III; **8**, Pachymic acid

Simultaneous quantitative and qualitative analyses of seven compounds in SGT were performed using HPLC–DAD and LC/MS, respectively. To improve the chromatographic separation capacity, 0.1% TFA (*v*/v) in water (A) and acetonitrile (B) were used as mobile phases in a gradient elution system. Furthermore, analyte resolution was found to be better with acetonitrile than with methanol. The present chromatographic conditions were used to establish the specific HPLC retention times (*t*_*R*_) and UV detection wavelengths for the seven standard compounds, which were used to identify the seven compounds in SGT and in one of the *Lactobacillus*-fermented SGT batches (SGT166). As shown in Fig. 2, the retention times of liquiritin, ginsenoside Rg_1_, liquiritigenin, glycyrrhizin, atractylenolide III, atractylenolide II, and atractylenolide Ι in the standard mixture were 15.25 (220 nm), 22.20 (200 nm), 23.91 (220 nm), 36.66 (254 nm), 44.76 (220 nm), 52.68 (220 nm), and 57.15 (275 nm) minutes, respectively. Under the same conditions, the retention times (min) of the observed components were 15.28 (liquiritin), 22.12 (ginsenoside Rg_1_), 23.95 (liquiritigenin), 36.59 (glycyrrhizin), 44.74 (atractylenolide III), 52.67 (atractylenolide II), and 57.14 (atractylenolide Ι) in SGT and 15.22 (liquiritin), 22.10 (ginsenoside Rg_1_), 23.95 (liquiritigenin), 36.55 (glycyrrhizin), 44.71 (atractylenolide III), 52.64 (atractylenolide II), and 57.11 (atractylenolide Ι) in SGT166. Pachymic acid as a standard compound of poria sclerotium was not detected in our optimized chromatographic method; therefore, pachymic acid was identified by LC/MS analysis (Fig. 3). In positive-ion mode, molecular ions for each compound in the standard mixture were observed at m/z 419.133 [M + H]^+^ for liquiritin, m/z 257.080 [M + H]^+^ for liquiritigenin, m/z 823.481 [M + Na]^+^ for ginsenoside Rg1, m/z 823.411 [M + H]^+^ for glycchirizine, m/z 231.137 [[M + H]^+^ for atractylenolide I, m/z 233.153 [M + H]^+^ for atractylenolide II, m/z 249.148 [M + H]^+^ for atractylenolide III, and m/z 529.388 [M + H]^+^ for pachymic acid. Under the same conditions, molecular ions for each components were detected at m/z 419.132 [M + H]^+^ for liquiritin, m/z 257.080 [M + H]^+^ for liquiritigenin, m/z 823.479 [M + Na]^+^ for ginsenoside Rg1, m/z 823.409 [M + H]^+^ for glycchirizine, m/z 231.137 [[M + H]^+^ for atractylenolide I, m/z 233.153 [M + H]^+^ for atractylenolide II, m/z 249.147 [M + H]^+^ for atractylenolide III, and m/z 529.387 [M + H]^+^ for pachymic acid in SGT; m/z 419.132 [M + H]^+^ for liquiritin, m/z 257.080 [M + H]^+^ for liquiritigenin, m/z 823.479 [M + Na]^+^ for ginsenoside Rg1, m/z 823.409 [M + H]^+^ for glycchirizine, m/z 231.137 [[M + H]^+^ for atractylenolide I, m/z 233.153 [M + H]^+^ for atractylenolide II, m/z 249.148 [M + H]^+^ for atractylenolide III, and m/z 529.388 [M + H]^+^ for pachymic acid in SGT166 (Additional file 1: Table S1).

### Analytical methods validation

To establish the calibration curve, mixed standard solutions of five different concentrations were analyzed in triplicate. Calibration curves were generated by plotting chromatographic peak area as a function of analyte concentration. Linear regressions of these data were expressed as *Y* = *Ax* + *B*, where *A* is the slope of the calibration curve, *B* is the y-intercept of the calibration curve, *x* is the concentration of marker components, and *Y* is the peak area. Correlation coefficients (*R*^*2*^) were calculated to assess linearity. The calibration data of each standard compound showed good linearity (*R*^*2*^ > 0.9999). The LOD and LOQ were determined at signal-to-noise (S/N) ratios of 3 and 10, respectively. The LOD was between 0.002 μg/mL and 0.023 μg/mL, and the LOQ was between 0.006 μg/mL^,^ and 0.069 μg/mL (Additional file 1: Table S2). The RSD (%) was < 3%, which indicated acceptable precision. The RSDs of the intra- and inter-day assays were between 0.02 and 1.80% and between 0.02 and 1.90%, respectively, with accuracy between 97.72 and 102.20% for the intra-day assay and between 98.11 and 103.06% for the inter-day assay. Accuracy was determined by a recovery test with an appropriate amount of SGT (10 mg/mL) that had been spiked with three different (low, medium, and high) quantities of authentic standards. The amounts of each of the seven compounds in the spiked SGT were calculated from the corresponding calibration curve. The results showed good accuracy with an overall recovery between 96.22 and 104.09% for the compounds concerned. These results demonstrated that the developed HPLC method was sufficiently accurate and sensitive for simultaneous quantitative evaluation of seven components from SGT (Additional file 1: Tables S3 and S4).

### Phytochemical characterization of SGT and FSGTs

The developed HPLC-based analytical method was applied to the simultaneous quantitation of seven components, including liquiritin, ginsenoside Rg_1_, liquiritigenin, glycyrrhizin, atractylenolide III, atractylenolide II, and atractylenolide Ι, in SGT and its FSGTs. Among them, the amounts of ginsenoside Rg_1_ in SGT and the FSGTs were lower than the LOQ even though Rg_1_was observed in HPLC chromatograms of SGT and FSGTs (Fig. 2). Therefore, it was recorded as not detected (nd). Except for ginsenoside Rg_1_, the six components in SGT and the FSGTs were identified by comparing their retention times and UV absorbances with those of the standard compounds. The contents of liquiritigenin, glycyrrhizin, and atractylenolide I–III were increased, whereas the content of liquiritin was decreased in the FSGTs relative to that of the SGT. These results indicate that the detected chemical components could be used as biomarkers for studying SGT and FSGTs.

In this study, following fermentation by all eight kinds of *Lactobacillus* strains, the conversion between liquiritin and liquiritigenin, an aglycone form of liquiritin, was clearly confirmed: the amount of liquiritigenin increased between 1275.84 and 1399.69%, and the amount of liquiritin decreased by 97.83 to 98.90%. Furthermore, glycyrrhizin contents in SGT was markedly changed by fermentation: the amount of glycyrrhizin increased between 199.44 and 279.28% (Table 2). In this study, fermentation of SGT by *L. plantarum* 166 (SGT166) showed distinct component changes among the eight kinds of *Lactobacillus* strains. As presented in Table 3, the conversion rates of liquiritigenin (1399.69%), glycyrrhizin (279.28%), atractylenolide III (29.96%), atractylenolide II (5.98%), and atractylenolide I (36.67%) in SGT166 were higher than those in SGT (100%).

**Table 2:** Amounts of the seven compounds in SGT and fermented SGTs (FSGTs)

Sample (*Lactobacillus* strains)	Amounts (mg/g)						
	Liquiritin	Ginsenoside Rg^1^	Liquiritigenin	Glycyrrhizin	Atractylenolide III	Atractylenolide II	Atractylenolide I
SGT	2.818	N.D.^a^	0.327	1.631	0.534	0.117	0.060
SGT127 (*L. rhamnosus*)	0.035	N.D.	4.709	4.884	0.696	0.123	0.079
SGT129 (*L. zeae*)	0.036	N.D.	4.718	5.720	0.695	0.130	0.079
SGT144 (*L. rhamnosus*)	0.034	N.D.	4.662	5.808	0.678	0.119	0.079
SGT150 (*L. acidophilus*)	0.031	N.D.	4.565	5.486	0.690	0.117	0.076
SGT162 (*L. fermentum*)	0.061	N.D.	4.499	5.044	0.673	0.123	0.079
SGT166 (*L. plantarum*)	0.034	N.D.	4.904	6.186	0.694	0.124	0.082
SGT217 (*L. acidophilus*)	0.031	N.D.	4.678	6.050	0.645	0.122	0.078
SGT341 (*L. helveticus*)	0.031	N.D.	4.472	5.920	0.696	0.126	0.079

^a^N.D., Not detected

**Table 3:** Conversion rate of detected six compounds in SGT116

Sample (strains)	Conversion rate (%)					
	Liquiritin	Liquiritigenin	Glycyrrhizin	Atractylenolide III	Atractylenolide II	Atractylenolide I
SGT	100.00	100.00	100.00	100.00	100.00	100.00
SGT166 (*Lactobacillus plantarum*)	−98.79	1499.69	379.28	129.96	105.98	136.67

### Comparison of the protective effects of SGT and SGT116 against toxicant-induced intracellular oxidative stress and disruption of MMPs in SH-SY5Y cells

Human neuroblastoma SH-SY5Y cells are widely used as an in vitro model in neuroscience research, including studies of neurobiology, neuronal differentiation, and neuroprotective events [21]. To determine the neuroprotective effects of SGT and SGT166, SH-SY5Y cells were treated with SGT or SGT166 at 250 μg/mL and 500 μg/mL for 24 h, respectively. The treatment with SGT or SGT166 had no significant cytotoxicity on SH-SY5Y cells; therefore, these concentrations were used in further studies (data not shown).

**Fig. 4:**
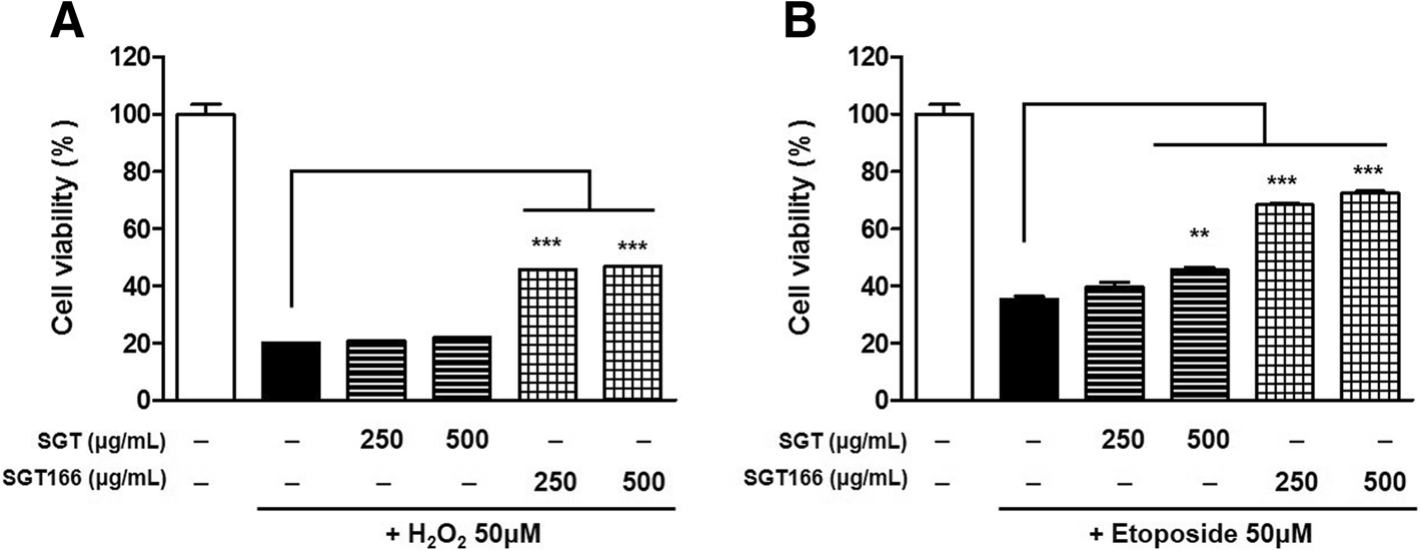
Neuroprotective effect of SGT and SGT166 against toxicant-induced SH-SY5Y cell loss. Cells were pretreated with SGT or SGT166 for 6 h, and then separately exposed to 50 μM H_2_O_2_ (**a**) and 50 μM etoposide (**b**) for 24 h. Cell viability was determined by CCK analysis and the results are expressed as the percentages of viable cells compared to the cells untreated with any toxicant. The values shown are means ± SE (*n* = 5). ^**^P < 0.01 and ^***^P < 0.001 versus the cells treated with toxicant only

**Fig. 5:**
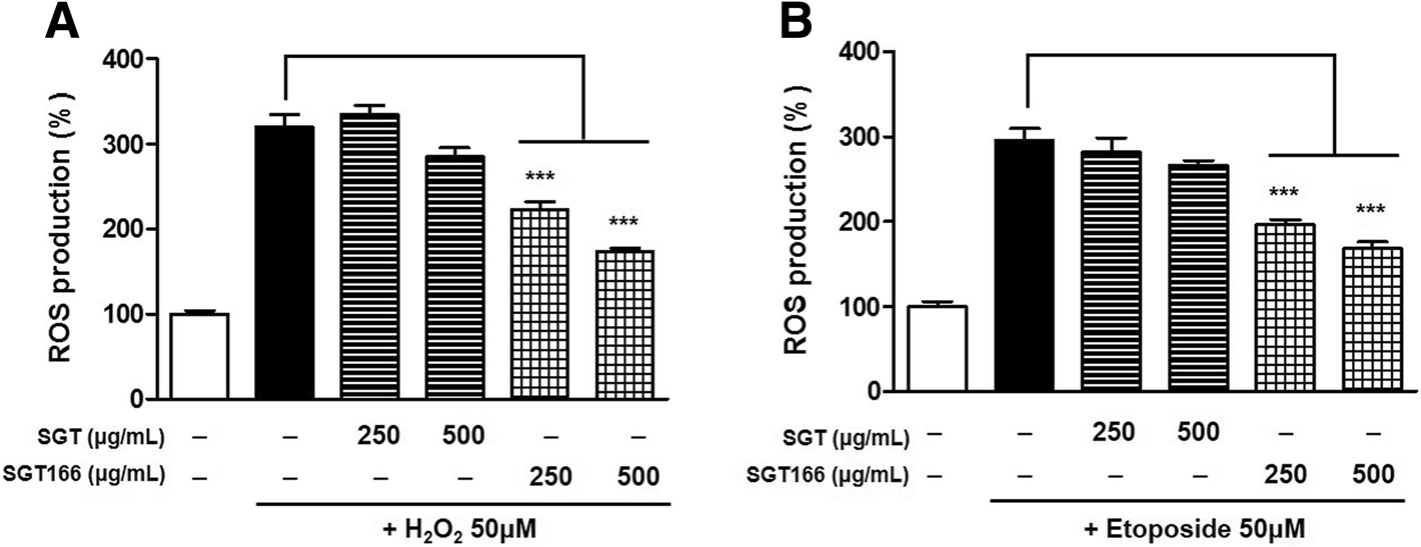
The inhibitory effects of SGT and SGT166 against toxicant-induced ROS production in SH-SY5Y cells. Total ROS levels were measured using the H_2_-DCFDA method. Cells were pretreated with SGT or SGT166 for 6 h, and then labeled with 50 μM H_2_-DCFDA for 30 min. Cells were separately exposed to 50 μM H_2_O_2_ (**a**) and 50 μM etoposide (**b**), analyzed immediately using a fluorescent plate reader. The values shown are means ± SE (*n* = 5). ^***^P < 0.001 versus the cells treated with toxicant only

**Fig. 6:**
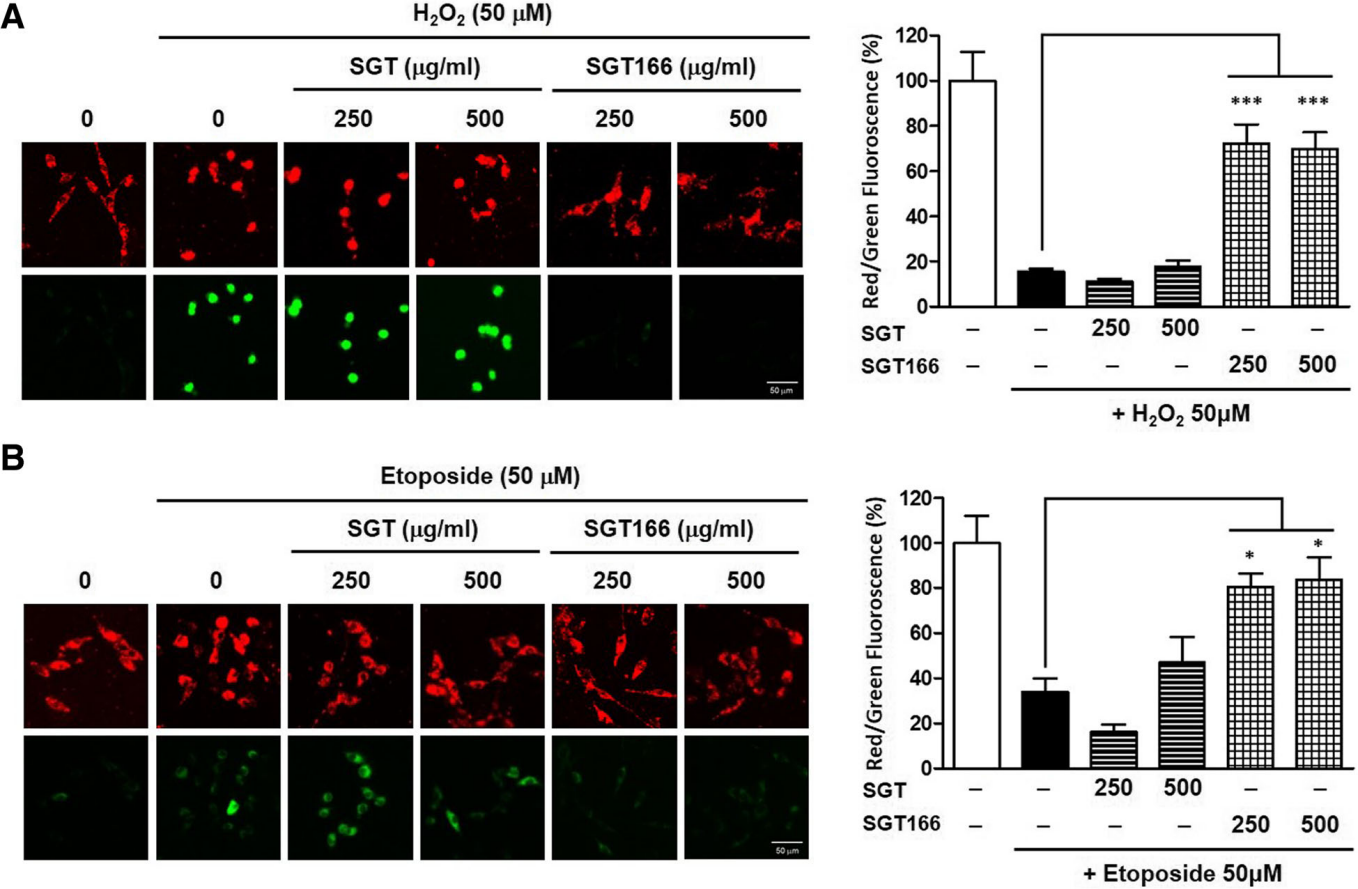
The protective effects of SGT and SGT166 against toxicant-induced disruption of MMP in SH-SY5Y cells. MMP was assessed by confocal microscopy using JC-1 staining. Cells were pretreated with SGT or SGT166 for 6 h, and then separately exposed to 50 μM H_2_O_2_ (**a**) and 50 μM etoposide (**b**) for 1 h. After incubated with JC-1 for 15 min, the cells were observed under 132x magnification. Representative images showing red fluorescence (aggregated form) and green fluorescence (monomeric form). Scale bar = 50 μm. The graph shows the red/green fluorescence intensity ratio quantitative analysis. The values shown are means ± SE (*n* = 5). ^*^P < 0.05 and ^***^P < 0.001 versus the cells treated with toxicant only

To access the neuroprotective effects of SGT or SGT166, H_2_O_2_ and etoposide were separately used to treat SH-SY5Y cells as a toxicant to induce ROS generation. The cells were pretreated with SGT or SGT166 for 6 h and then separately exposed to 50 μM H_2_O_2_ and 50 μM etoposide for 24 h. CCK analysis showed that SGT166 significantly protected toxicant-induced SH-SY5Y cell loss, whereas SGT had a much lesser effect (Fig. 4). To further investigate the effect on endogenous ROS in SH-SY5Y cells, SGT and SGT166 were treated separately with 50 μM H_2_O_2_ or 50 μM etoposide. After 24 h, the levels of intracellular ROS were measured using H_2_-DCFDA, a fluorescent dye oxidized to fluorescent DCF by ROS. Toxicant treatment caused a marked increase in intracellular ROS generation, and SGT166 pretreatment significantly reduced ROS production, whereas SGT showed a negligible blocking of ROS production (Fig. 5). For this reason, the protective effects of SGT and SGT166 against toxicant-induced disruption of MMP in SH-SY5Y cells were evaluated using JC-1 staining. As shown in Fig. 6, treatment with 50 μM H_2_O_2_ or 50 μM etoposide decreased red fluorescence and increased green fluorescence in the cells, which indicated MMP loss. Particularly, 250 μg/mL SGT166 protected against MMP disruption induced by all toxicants in SH-SY5Y cells, which showed approximately three-times significantly greater activity than that of SGT.

## Discussion

*Lactobacillus* strains are components of the intestinal microflora in humans and known to be associated with the health of the host [22, 23]. It has also been reported that *Lactobacillus* strains exhibit glycosidase activity and have a critical role in the intestinal hydrolysis of various plant glycosides [24]. Cho et al. reported on the hydrolysis of daidzin and genistin to their respective aglycones, daidzein and genistein, by the *Lactobacillus* strains. The concentrations of daidzein and genistein increased by 910 and 830%, respectively, following fermentation [25]. As a major component of glycyrrhizae radix, glycyrrhizin is often referred to by chemists as glycyrrhizic acid. Glycyrrhizin is metabolized to glycyrrhizic acid, a major physiologically active ingredient, by β-D-glucuronidase in vivo [26]. The carboxyl group in glycyrrhizin is representative of the acidic substances produced by *Lactobacillus* strains during fermentation in a reduced pH environment [27]. In our previous study, conversion from liquiritin to liquiritigenin and an increase in glycyrrhizin contents caused by *Lactobacillus* bacteria was observed in fermented Yijin-tang, a traditional prescription [28]. However, the greatest fermentation efficacy was observed for YJ221 fermented by *L. brevis,* not for YJ166 fermented by *L. plantarum*. These results suggest that the optimal *Lactobacillus* strain could be applied to achieve conversion of specific phytochemicals in herbal medicine and that *L. plantarum* 166 might be the optimal *Lactobacillus* strain for fermenting SGT.

Oxidative stress is involved in cell proliferation, differentiation, and survival by activating signaling pathways; however, persistent or high levels of oxidative stress cause neurotoxicity in neurodegenerative diseases [15, 29]. Mitochondrial permeability is associated with cell toxicity, oxidative damage, and apoptosis. ROS causes mitochondrial dysfunction through mitochondrial matrix swelling and outer membrane rupture, which ultimately triggers ROS-dependent mitochondrial apoptosis [19, 20]. Aggregated JC-1 exhibits red fluorescence in healthy mitochondria, whereas the monomeric form is characterized by green fluorescence when mitochondria are depolarized during apoptotic cell death. The intensity ratio of red/green fluorescence reflects the degree of intact mitochondria. In this study, the pretreatment of SGT had a very low preventive effect against the MMP loss caused by H_2_O_2_ or etoposide even though some activity was observed at high concentrations (500 μg/mL) relative to that of toxicant-treated cells. In contrast, SGT166 pretreatment strongly prevented the loss of MMP by H_2_O_2_ or etoposide, which demonstrates that the changed phytochemicals in SGT166 by fermentation are involved in improving neuroprotective effect.

In the HPLC analysis of SGT and SGT166, liquiritigenin, glycyrrhizin, and atractylenolide I–III were increased by *Lactobacillus* fermentation. Previous studies have reported that an ethanol extract of Glycyrrhizae radix containing liquiritigenin and glycyrrhizin prevented amyloid beta-induced neuronal cell death by interfering with ROS [30]. Atractylodis rhizome and its active components, atractylenolide I and III, also have been reported to exert a neuroprotective effect against neuronal apoptosis [31–33]. Recently, atractylenolide I was shown to exert significant anti-neuroinflammatory activities in vitro and in a Parkinson’s disease model in vivo [34]. This evidence supports the hypothesis that an increase in active compounds caused by fermentation of SGT might strongly contribute to the neuroprotective effect in SH-SY5Y human neuroblastoma. In addition, these results support that inhibiting ROS generation may be one of the neuroprotective actions of SGT166.

## Conclusion

This study demonstrated that *Lactobacillus* fermentation enhanced the specific effects of an herbal medicine, SGT. Especially, SGT166, a SGT fermented with *L. plantarum* 166, enhanced neuroprotective effects by suppressing oxidative stress and MMP loss. The enhancement was thought to be caused by conversion of phytochemicals during the fermentation. Therefore, SGT166 is a potential candidate for treatment of neurological damage-related diseases. The study results should be complemented by investigations of the functional mechanisms of SGT166 in neuroblastoma and evaluation of the neuroprotective effect in an in vivo model.

## Additional file

Additional file 1: **Table S1.** Calibration curves, limit of detection (LOD), and limit of quantification (LOQ) of the seven compounds. **Table S2.** Precision (intra- and interday) and accuracy of the seven compounds. **Table S3.** The percent recovery of the seven compounds. **Table S4.** Identification of the phytochemicals in SGT and SGT166 by UPLC-ESI-MS analysis. (DOCX 31 kb)

## Abbreviations

AD: Alzheimer’s disease
ALS: Amyotrophic lateral sclerosis
CCK-8: Cell counting kit-8
FSGTs: Fermented SGTs
H_2_-DCFDA: 2′, 7′-dichlorodihydrofluorescein diacetate
HPLC-DAD: High-performance liquid chromatography with diode-array detection
ICH: International conference on harmonization
LC/MS: Liquid chromatography/mass spectrometry
LOD: Linearity, limit of detection
LOQ: Limit of quantification
MMPs: mitochondrial membrane potentials
MS: Mutiple Sclerosis
PD: Parkinson’s disease
RNS: Reactive nitrogen species
ROS: Reactive oxygen species
RSD: Relative standard deviation
SGT: Sagunja-tang
TFA: Trifluoroacetic acid
YJ: Yijin-tang
