# Supplementary material for: Enhancement of neuroprotective activity of Sagunja-tang by fermentation with lactobacillus strains
Source: BMC Complement Altern Med. 2018 Nov 28;18:312. doi: 10.1186/s12906-018-2361-z (PMC6263064; doi:10.1186/s12906-018-2361-z)
Supplement: Supplementary file 1 — Table S1. Calibration curves, limit of detection (LOD), and limit of quantification (LOQ) of the seven compounds. Table S2. Precision (intra- and interday) and accuracy of the seven compounds. Table S3. The percent recovery of the seven compounds. Table S4. Identification of the phytochemicals in SGT and SGT166 by UPLC-ESI-MS analysis. (DOCX 31 kb) [file 12906_2018_2361_MOESM1_ESM.docx]

Additional file 1: Table S1. Identification of the phytochemicals in SGT and SGT166 by UPLC-ESI-MS analysis

| No | | t_R_ (min) | Precursor ion (m/z) | Estimated | Calculated | Elemental composition | Error  (ppm) | Identification* |
| --- | --- | --- | --- | --- | --- | --- | --- | --- |
| **SGT** | | |  |  |  |  |  |  |
| 1 | 10.55 | | [M+H]^+^ | 419.13366 | 419.13275 | C_21_H_22_O_9_ | -2.16765 | Liquritin |
| 2 | 13.69 | | [M+H]^+^ | 257.08084 | 257.08029 | C_15_H_12_O_4_ | -2.13261 | Liquiritigenin |
| 3 | 13.19 | | [M+Na]^+^ | 823.48143 | 823.47980 | C_42_H_72_O_14_ | -1.98260 | Ginsenoside Rg1 |
| 4 | 17.91 | | [M+H]^+^ | 823.41106 | 823.40955 | C_42_H_62_O_16_ | -1.83882 | Glycchyrizin |
| 5 | 22.21 | | [M+H]^+^ | 231.13796 | 231.13766 | C_15_H_18_O_2_ | -1.27718 | Atractylenolide I |
| 6 | 21.19 | | [M+H]^+^ | 233.15361 | 233.15321 | C_15_H_20_O_2_ | -1.70059 | Atractylenolide II |
| 7 | 19.58 | | [M+H]^+^ | 249.14852 | 249.14793 | C_15_H_20_O_3_ | -2.35217 | Atractylenolide III |
| 8 | 24.11 | | [M+H]^+^ | 529.38875 | 529.38763 | C_33_H_52_O_5_ | -2.10757 | Pachymic acid |
| **SGT166** |  | |  |  |  |  |  |  |
| 1 | 10.51 | | [M+H]^+^ | 419.13366 | 419.13272 | C_21_H_22_O_9_ | -2.24046 | Liquritin |
| 2 | 13.69 | | [M+H]^+^ | 257.08084 | 257.08029 | C_15_H_12_O_4_ | -2.13261 | Liquiritigenin |
| 3 | 13.17 | | [M+Na]^+^ | 823.48143 | 823.47949 | C_42_H_72_O_14_ | -2.35320 | Ginsenoside Rg1 |
| 4 | 17.90 | | [M+H]^+^ | 823.41106 | 823.40930 | C_42_H_62_O_16_ | -2.13532 | Glycchyrizin |
| 5 | 22.21 | | [M+H]^+^ | 231.13796 | 231.13750 | C_15_H_18_O_2_ | -2.00336 | Atractylenolide I |
| 6 | 21.19 | | [M+H]^+^ | 233.15361 | 233.15323 | C_15_H_20_O_2_ | -1.63515 | Atractylenolide II |
| 7 | 19.61 | | [M+H]^+^ | 249.14852 | 249.14804 | C_15_H_20_O_3_ | -1.92347 | Atractylenolide III |
| 8 | 24.12 | | [M+H]^+^ | 529.38875 | 529.38867 | C_33_H_52_O_5_ | -0.14758 | Pachymic acid |

*, compared with the retention time and MS spectral data of an authentic standards

Additional file 1: Table S2. Calibration curves, limit of detection (LOD), and limit of quantification (LOQ) of the seven compounds

| Compounds | Linear range (µg/ml) | Regression equation^a^ | Correlation coefficient (*R*^2^) | LOD (µg/ml) | LOQ (µg/ml) |
| --- | --- | --- | --- | --- | --- |
| Liquiritin | 0.625 ~ 50.000 | y = 58993.75*x* + 2082.65 | 1.0000 | 0.023 | 0.069 |
| Ginsenoside Rg_1_ | 0.781 ~ 62.500 | y= 3592.63*x* - 159.92 | 0.9999 | 0.020 | 0.060 |
| Liquiritigenin | 0.312 ~ 25.000 | y = 54207.02*x* - 1022.20 | 1.0000 | 0.011 | 0.033 |
| Glycyrrhizin | 1.406 ~ 112.500 | y = 10270.69*x* + 960.39 | 1.0000 | 0.014 | 0.044 |
| Atractylenolide III | 0.312 ~ 25.000 | y = 50940.34*x* - 584.75 | 1.0000 | 0.008 | 0.023 |
| Atractylenolide II | 0.312 ~ 25.000 | y = 79496.11*x* - 1039.86 | 1.0000 | 0.002 | 0.006 |
| Atractylenolide I | 0.312 ~ 25.000 | y = 89749.65*x* + 4497.85 | 1.0000 | 0.019 | 0.059 |

Additional file 1: Table S3 Precision (intra- and interday) and accuracy of the seven compounds

| Compounds | Concentration  (μg/ml) | Intra- day (n=5) | | |  | Inter- day (n=5) | | |
| --- | --- | --- | --- | --- | --- | --- | --- | --- |
|  |  | Mean ± SD (μg/ml) | RSD (%) | Accuracy (%) |  | Mean ± SD (μg/ml) | RSD (%) | Accuracy (%) |
| Liquiritin | 25.00 | 24.72 ± 0.01 | 0.03 | 98.86 |  | 24.68 ± 0.08 | 0.31 | 98.73 |
|  | 12.50 | 12.28 ± 0.01 | 0.07 | 98.27 |  | 12.31 ± 0.02 | 0.17 | 98.49 |
|  | 6.25 | 6.11 ± 0.01 | 0.08 | 97.72 |  | 6.13 ± 0.01 | 0.10 | 98.11 |
| Ginsenoside Rg_1_ | 31.25 | 31.45 ± 0.24 | 0.76 | 100.65 |  | 32.21 ± 0.61 | 1.90 | 103.06 |
|  | 15.63 | 15.69 ± 0.28 | 1.80 | 100.44 |  | 15.49 ± 0.06 | 0.39 | 99.16 |
|  | 7.81 | 7.78 ± 0.08 | 1.09 | 99.61 |  | 7.74 ± 0.05 | 0.64 | 99.04 |
| Liquiritigenin | 12.50 | 12.31 ± 0.01 | 0.06 | 98.49 |  | 12.33 ± 0.01 | 0.04 | 98.63 |
|  | 6.25 | 6.32 ± 0.01 | 0.04 | 101.10 |  | 6.31 ± 0.01 | 0.10 | 100.91 |
|  | 3.13 | 3.07 ± 0.01 | 0.19 | 98.30 |  | 3.07 ± 0.01 | 0.16 | 98.30 |
| Glycyrrhizin | 56.25 | 55.91 ± 0.06 | 0.11 | 99.39 |  | 55.91 ± 0.09 | 0.15 | 99.40 |
|  | 28.13 | 27.90 ± 0.03 | 0.10 | 99.21 |  | 27.91 ± 0.01 | 0.03 | 99.25 |
|  | 14.06 | 13.83 ± 0.05 | 0.37 | 98.34 |  | 13.86 ± 0.02 | 0.14 | 98.59 |
| Atractylenolide III | 12.50 | 12.30 ± 0.01 | 0.09 | 98.44 |  | 12.31 ± 0.01 | 0.03 | 98.51 |
|  | 6.25 | 6.15 ± 0.01 | 0.09 | 98.36 |  | 6.17 ± 0.01 | 0.07 | 98.62 |
|  | 3.13 | 3.07 ± 0.01 | 0.19 | 98.17 |  | 3.07 ± 0.01 | 0.28 | 98.28 |
| Atractylenolide II | 12.50 | 12.58 ± 0.01 | 0.06 | 100.60 |  | 12.56 ± 0.01 | 0.03 | 100.34 |
|  | 6.25 | 6.26 ± 0.01 | 0.12 | 100.22 |  | 6.26 ± 0.01 | 0.06 | 100.18 |
|  | 3.13 | 3.12 ± 0.01 | 0.04 | 99.75 |  | 3.12 ± 0.01 | 0.06 | 99.69 |
| Atractylenolide I | 12.50 | 12.69 ± 0.01 | 0.06 | 101.50 |  | 12.67 ± 0.01 | 0.11 | 101.38 |
|  | 6.25 | 6.39 ± 0.01 | 0.02 | 102.20 |  | 6.38 ± 0.02 | 0.26 | 102.04 |
|  | 3.13 | 3.10 ± 0.01 | 0.07 | 99.23 |  | 3.10 ± 0.01 | 0.02 | 99.20 |

Additional file 1: Table S4. The percent recovery of the seven compounds

| Compounds | Spiked Amount  (μg/ml) | Measured Amount  (μg/ml) | RSD^a^  (%) | Recovery^b^  (%) |
| --- | --- | --- | --- | --- |
| Liquiritin | 12.50 | 12.65 ± 0.03 | 0.26 | 101.23 |
|  | 6.25 | 6.46 ± 0.07 | 1.11 | 103.32 |
|  | 3.13 | 3.07 ± 0.01 | 0.38 | 98.20 |
| Ginsenoside Rg1 | 15.63 | 15.03 ± 0.22 | 1.47 | 96.22 |
|  | 7.81 | 7.87 ± 0.09 | 1.20 | 100.79 |
|  | 3.91 | 4.00 ± 0.09 | 2.35 | 102.36 |
| Liquiritigenin | 6.25 | 6.20 ± 0.02 | 0.29 | 99.18 |
|  | 3.13 | 3.17 ± 0.01 | 0.24 | 101.55 |
|  | 1.56 | 1.58 ± 0.01 | 0.53 | 101.18 |
| Glycyrrhizin | 28.13 | 28.71 ± 0.14 | 0.47 | 102.09 |
|  | 14.06 | 14.64 ± 0.08 | 0.57 | 104.09 |
|  | 7.03 | 6.94 ± 0.09 | 1.31 | 98.66 |
| Atractylenolide III | 6.25 | 6.36 ± 0.03 | 0.53 | 101.81 |
|  | 3.13 | 3.06 ± 0.01 | 0.16 | 98.01 |
|  | 1.56 | 1.53 ± 0.01 | 0.47 | 98.02 |
| Atractylenolide II | 6.25 | 6.20 ± 0.01 | 0.21 | 99.13 |
|  | 3.13 | 3.12 ± 0.01 | 0.19 | 99.85 |
|  | 1.56 | 1.54 ± 0.01 | 0.16 | 98.33 |
| Atractylenolide I | 6.25 | 6.15 ± 0.01 | 0.10 | 98.34 |
|  | 3.13 | 3.22 ± 0.01 | 0.13 | 103.09 |
|  | 1.56 | 1.61 ± 0.01 | 0.28 | 102.73 |

^a^RSD, relative standard deviation; ^b^Recovery (%) = [(found–original) / spiked] × 100%
